# Outcomes of deep brain stimulation surgery in the management of dystonia in glutaric aciduria type 1

**DOI:** 10.1007/s00415-025-12942-3

**Published:** 2025-03-01

**Authors:** Daniel E. Lumsden, Stavros Tsagkaris, Jon Cleary, Michael Champion, Helen Mundy, Abteen Mostofi, Harutomo Hasegawa, Verity M. McClelland, Shakya Bhattacharjee, Monty Silverdale, Hortensia Gimeno, Keyoumars Ashkan, Richard Selway, Margaret Kaminska, Alexander Hammers, Jean-Pierre Lin

**Affiliations:** 1 Complex Motor Disorder Service, Evelina London Children’s Hospital, Guy’s and St Thomas’ NHS Foundation Trust, Floor 2, Beckett House, Westminster Bridge Road, London, SE1 7DB UK; 2 Research Department of Early Life Imaging, Biomedical Engineering and Imaging Sciences, King’s College London, London, UK; 3 King’s College London and Guy’s and St Thomas’ PET Centre, Research Department of Biomedical Computing, School of Biomedical Engineering and Imaging Sciences, King’s College London, London, UK; 4 Neuroradiology, Department of Radiology, Guy’s and St Thomas’ NHS Foundation Trust, London, UK; 5 School of Biomedical Engineering and Imaging Sciences, King’s College London, London, UK; 6 Inherited Metabolic Disease, Evelina London Children’s Hospital, Guy’s and St Thomas’ NHS Foundation Trust, London, UK; 7 Functional Neurosurgery, King’s College Hospital, London, UK; 8 Department of Basic and Clinical Neuroscience, Institute of Psychiatry, Psychology and Neuroscience, King’s College London, London, SE5 8AF UK; 9 Department of Clinical Neurophysiology, Guy’s and St Thomas’ NHS Foundation Trust, London, UK; 10 Neurology, Queen Elizabeth Hospital, University Hospital Birmingham NHS Foundation Trust and Russells Hall Hospital, Dudley Group Foundation Trust, Birmingham, UK; 11 Department of Neurology and Neurosurgery, Salford Royal Hospital NHS Foundation Trust, Salford, UK; 12 Barts NHS Health and Queen Mary University of London, Wolfson Institute of Population Health, Centre for Preventive Neurology, London, UK; 13 Department for Women and Children, Faculty of Life Sciences and Medicine, Kings College London, London, UK

**Keywords:** Dystonia, Glutaric Aciduria, Deep Brain Stimulation, FDG-PET

## Abstract

**Objectives:**

Glutaric aciduria type 1 (GA1) is a rare autosomal recessive organic acidaemia caused by deficiency of the glutaryl-CoA dehydrogenase enzyme. We describe the outcomes following deep brain stimulation (DBS) for the management of dystonia of children and adults with glutaric aciduria type 1 (GA1).

**Methods:**

Cases with GA1 were identified from the institutional databases of two tertiary movement disorder services. Data were extracted from clinical records using a standardised proforma, including baseline clinical characteristics, imaging and neurophysiological findings, complications post-surgery, and outcomes as measured by the Burke–Fahn–Marsden Dystonia Rating Scale (BMFDRS) motor scores and the Canadian Occupation Performance Measure (COPM).

**Results:**

A total of 15 children were identified aged 3–17.5 with a median age of 11.5 years at neurosurgery, and one adult undergoing DBS aged 31 years. Baseline BMFDRS motor score ranged from 58.5–114, median 105. GMFCS-equivalence level was 5 (i.e. non-ambulant) for 10/16 cases. Surgery was tolerated in all cases without evidence of metabolic decompensation. BFMDRS motor score 1-year post-surgery ranged from 57.5–108.5 (median 97.25) and at last follow-up 57.5–112 (median 104) (no statistically significant change compared to baseline at either time point, *P* > 0.05). COPM data were available for 11/13 children and young people (CAYP). Clinically significant improvement was reported in 7/11 at 1 year and 8/11 at last follow-up. Four CAYP transitioned to adult services. Death occurred in three cases during follow-up, in no case related to DBS.

**Conclusion:**

DBS may be considered as a management option for children with GA1 who have appropriately selected goals for intervention.

## Introduction

First described in 1975, glutaric aciduria (GA1) is an autosomal-recessive organic acidaemia due to deficiency of glutaryl-CoA dehydrogenase (GCDH) which results in abnormal metabolism of lysine, hydroxylysine and tryptophan [5]. GA1 affects 1 in 100,000 newborns [24]. Children and young people (CAYP) with GA1 are usually asymptomatic until the development of an acute encephalopathic crisis between the age of 2 and 36 months. Catabolic episodes, often triggered by a febrile illness, give rise to these crises, following which striatal injury results in a complex motor disorder with prominent dystonia [21, 25]. For 10–20% of patients with GA1, in the absence of an acute decompensation there is an insidious onset of a movement disorder, typically resulting in less severe dystonia [3]

Dystonia in CAYP is intrusive, impairing function, interfering with the delivery of daily care, and causing pain [30]. Pharmacological interventions offer limited efficacy in the management of childhood dystonia and, even when effective, use is often limited by significant side effects [32]. Consequently, there has been major interest in the past 20 years in the application of deep brain stimulation (DBS) for CAYP with dystonia [8–10, 12]. A recent meta-analysis of 321 children undergoing DBS reported improvement in dystonic symptoms in 86.3% of cases [12], highlighting the potential benefits of this intervention.

Dystonia in CAYP is aetiologically heterogenous [28], and data on outcomes following DBS for CAYP with rare causes of dystonia such as GA1 are often limited.

We have previously reported the short-term outcomes of three CAYP with GA1 undergoing bilateral pallidal DBS for the management of their medication-refractory movement disorder [31]. Only six other single case reports have been published [1, 7, 29, 33, 39, 44]. To the best of our knowledge, a total of only nine patients with outcome data following DBS in GA1 have been reported to date.

The aim of this study is to expand upon published data by presenting a retrospective analysis of CAYP and adults with GA1 undergoing DBS at two institutions, including baseline clinical characteristics, response to DBS and reported complications.

## Methods

This was a retrospective analysis of imaging and assessments performed as part of standard clinical practise, and thus formal ethical approval was not required under National Health Service (NHS) research governance arrangement. All families gave written consent for imaging and surgical procedures.

### Patient ascertainment

Individuals with a confirmed diagnosis of GA1 undergoing DBS between July 2005 and July 2022 were identified from the institutional database at the Evelina London Children’s Hospital (ELCH), Guy’s and St Thomas’ NHS Foundation Trust, London UK. One additional case, undergoing surgery in adulthood, at the Salford Royal University Hospital (SRUH) NHS Foundation Trust, was also included (Case 16). In all cases, a diagnosis of GA1 had been made on the basis of biochemical testing for elevations in plasma glutaric acid, 3-hydroxyglutaric acid, glutaconic acid, and glutarylcarninitine, also confirmed with GCDH gene analysis.

### Clinical assessment

Demographical and clinical data were extracted for each individual identified, including clinical onset, age at surgery, baseline measures of functional ability (Gross Motor Function Classification System (GMFCS) level equivalent [40] and Manual Ability Classification System (MACS) [11] level equivalent), and baseline dystonia severity as measured by the Burke–Fahn–Marsden Dystonia Rating Scale (BFMDRS) [6]

All CAYP at ELCH had medication-refractory generalised dystonia leading to consideration of DBS surgery, suitability for which was assessed by an experienced multi-disciplinary team. Case 16 underwent surgery in adulthood following assessment of suitablility for DBS by the multi-disciplinary team SRUH.

### FDG-PET-CT image acquisition

A total of 14/16 cases underwent resting FDG-PET CT imaging prior to surgery. FDG-PET imaging provides information as to the metabolic activity of brain tissue and has been routinely used as part of the assessment process of CAYP undergoing evaluation as potential candidates for DBS surgery at the ELCH. FDG-PET imaging can help with the qualitative assessment of the target nuclei for DBS insertion, particularly where structural neuroimaging demonstrates areas of brain injury. As previously described, [^18^F]2-fluoro-2-deoxy-d-glucose (FDG)-PET imaging has been used to help assess eligibility for DBS surgery since 2005 at the ELCH. Prior to October 2013 all CAYP underwent FDG-PET-CT imaging on a GE (General Electric Medical Systems, Waukesha, WI) Discovery ST and a Discovery VCT scanner. Thereafter, scans were conducted using a GE Discovery 710 scanner at the King’s College London and Guy’s and St Thomas’ PET Centre. The FDG dose injected was scaled relative to a 250-MBq dose for a 70-kg adult as 250/70*child’s weight [kg]. FDG was injected after a 3-h fast and followed by a 30-min uptake period in a quiet room. Brief general anaesthesia was then induced, only for the duration of the 15-min PET-CT image acquisition, to eliminate dystonia-related motion artefact during scanning. General anaesthesia was initiated after the uptake period so FDG uptake reflects brain metabolism during wakefulness. For one case (Case 1 in Table 1) a continuous infusion of intravenous 10% dextrose was delivered during FDG-PET acquisition.

**Table 1:** Clinical characteristics and outcome findings

Case number	Mode of clinical onset (age of onset)	Age at surgery (years)	GMFCS	MACS	Follow-up years	Status	BFMDRS baseline	BFMDRS 1 year	BFDMRS last follow-up	COPM 1 year	COPM last follow-up	Comments
1	Decompensation (5 months)	10.6	5	5	1	Died	112	95.5	95.5	NA	NA	Episode status Dystonicus 6 months post-implantation. Died 18 months later unrelated to DBS
2	Decompensation (5 months)	15.7	5	5	2	Transition	114	105.5	104.5	NA	NA	
3	Decompensation (5 months)	11.8	5	5	8	Transition	110	104	112	Significant improvement	Significant improvement	
4	Screened as affected sibling (3 months)	19	5	5	2/9^a^	Died (following transition)	108.5	104	104	Significant improvement	Significant improvement	DBS system removed due to infection 10 years post-implantation
5	Decompensation (3 months)	13.25	4	4	6/3	Transition—active follow-up	94.5	95	99	No significant improvement	Significant improvement	Revision of right electrode 3.25 years post-implantation. Following transition, battery change performed 7 years post-implantation, with subsequent infection requiring repositioning of the implant, and then repositioning of a “flipped” battery 8 years post-implantation
6	Insidious (30 months)	6.5	4	4	5	Active follow-up	74	65	65	Significant improvement	No significant improvement	
7	Decompensation (10 months)	8.25	5	5	1	Explanted Due to Infection	95	NA	NA	NA	NA	
8	Decompensation (9 months)	4	5	5	2	Active follow-up	105.5	98.5	105.5	Significant improvement	No sgnificant improvement	
9	Decompensation (9 months)	4.25	5	5	2	Active follow-up	108.5	106.5	111.5	Significant improvement	Significant improvement	
10	Decompensation (8 months)	16.33	4	5	2	Active follow-up p	104	108.5	108.5	Significant improvement	Significant improvement	
11	Decompensation (8 months)	11.25	2	3	2	Active follow-up	58.5	57.5	57.5	No significant improvement	Significant improvement	
12	Insidious (1 month)	7.5	5	5	0	Left UK	114.5	NA	NA	NA	NA	Family emigrated/lost to follow-up
13	Decompensation (3 months)	14.8	4	3	2	Active follow-up	76.5	75	76	Significant improvement	Significant improvement	
14	Decompensation (5 months)	16.1	5	5	1	Active follow-up	102.5	NA	NA	No Significant improvement	No significant improvement	Loose connector revised 3 months post-implantation, then revision electrode 13 months post-implantation
15	Decompensation (7 months)	3	5	5	5	Died	105	96	108	Significant improvement	Significant improvement	System revised 5 years post-implantation. Died 3 months later unrelated to DBS
16^b^	Decompensation (< 1 year)	30	5	5	9	Active follow-up	NA	NA	NA	NA	NA	

Changes in COPM were considered clinically significant if there was a 2 point or greater change in either COPM performance or COPM satisfaction score

*BFMDRS* Burke–Fahn–Marsden Dystonia Rating Scale, *COPM* Canadian Occupational Performance Measure, *GMFCS* Gross Motor Function Classification System level, *MACS* Manual Ability Classification System, *NA* not available

^a^Data follow-up in years pre/post transition to adult services

^b^Case operated on at Salford Royal University Hospital Trust

### Structural imaging assessment

During the 17 years over which data were reviewed, MRI sequences routinely acquired as part of pre-operative work-up evolved, with acquisition on 1.5 T General Electric or Siemens scanners, or more recently a 3 T Siemens scanner. In all cases T_1_-, T_2_- and proton density weighted sequences were available for assessment for cases operated at the ELCH. Available pre-operative MRI images were reviewed and assessed for the presence of regional abnormalities (signal change, and/or volume loss) including in the pallidum, putamen, caudate or thalamus by an experienced Consultant Neuroradiologist (author JC). Each of these regions was classified as "normal" or "abnormal", as was the white matter.

### Pre-operative neurophysiological assessment

Central motor conduction times (CMCT) and somatosensory evoked potentials (SEPs) were obtained as part of the pre-operative assessment at ELCH and analysed as previously reported [34, 35]. CMCT and SEP measurements provide information on the intergrity of major motor and sensory pathways in the brain. We have previously demonstrated in a cohort of CAYP undergoing assessment for DBS that integrity of these pathways may be demonstrated in CAYP for whom MRI neuroimaging might suggest white matter or other brain injury which would potentially preclude the application of DBS [34]. Furthermore, abnormalities in either CMCT or SEP recordings predicts a poorer outcome for CAYP following DBS [35] CMCT recordings were available for all children, but as SEPs were added to routine patient assessment later than CMCTs, they were not recorded in all cases. CMCTs and SEPs were recorded from all four limbs with each patient classified as having “abnormal” testing if the recordings from one or more limbs were abnormal [34, 35].

### Peri-operative metabolic management

Individualised peri-operative metabolic management plans were followed in line with British Inherited Metabolic Disease Group guidance [19].

### Surgical procedure

For all cases undergoing surgery following assessment at the ELCH, the surgery entailed bilateral implantation of quadripolar DBS electrodes (Medtronic Model 3389) with direct MRI-guided targeting technique under general anaesthesia and with the use of the Leksell frame (pre-2016) or the Neuromate (Renishaw, UK) robot (post-2016). Intraoperative microelectrode recording [36] was performed for most cases until 2016. Lead placement was confirmed by intra-operative CT imaging for cases up to 2019, following which the O-arm intra-operative imaging system was used. DBS programming was initiated on the day of surgery by a movement disorder paediatric neurologist. For Case 16 electrode placement was confirmed with peri-operative MRI. In all cases bilateral DBS was performed, targeting the GPi. All cases operated on at the ELCH received Medtronic Activa RC neurostimulators, except Case 1 (bilateral Medtronic Soletra neurostimulators) and Case 2 (Medtronic Kinetra neurostimulator). Case 16 received a Boston Scientific Vercise neurostimulator.

### DBS programming

DBS programming for the ELCH cohort followed a previously described progression [31]. Pulse generators were initially programmed with a voltage of 0.5 V, pulse width of 450 μs, and stimulation frequency of 130 Hz, with bilateral single monopolar contacts. Based on clinical response, voltages were then increased over subsequent months as indicated and tolerated, before pragmatic activation of second, third and, much less commonly, a fourth monopolar contact. For Case 16 a similar stepwise approach was used, with stimulation initiated 6–8 weeks post-operatively, but with a starting pulse width of 60 microseconds.

### Outcome measures following surgery

For CAYP from ELCH, outcome following DBS was assessed using two standardised measures: the percentage change in BMFDRS and the change in Canadian Occupational Performance Measure (COPM) [26]. Both were conducted by highly specialised therapists within the ELCH multi-disciplinary team. BFMDRS motor scores assessed at baseline, 1-year post-surgery and at last available follow-up were collected, with percentage change from baseline BMFDRS score calculated at each time-point. Where available, change in Canadian Occupational Performance Measure (COPM) [26] at these time points was also collected. The COPM is a client-centred tool used for CYP and their carers, to identify what daily life problem areas to address with interventions, setting personalised goals. Each “goal” is scored at baseline and following intervention, with a change in score of two points averaged over 5 goal areas being considered clinically relevant [27]. Complications following surgery were identified for each CAYP from a prospectively maintained database [23]. Standardised measures were not recorded for Case 16.

## Results

### Clinical cases

From a total 235 CAYP undergoing primary DBS implantation at the ELCH over the study time period, 15 CAYP with GA1 were identified, representing 6.4% of the implanted population. One additional case operated on in adulthood at the SRUH was also included i.e. a total of 16 cases. Clinical details for these participants are summarised in Table 1. Short-term outcomes for cases 1–3 have been previously reported [31]. CAYP ranged in age from 3–17.5 (median 11.25 years) at the time of surgery. Case 16 was 31 years old at the time of surgery. In 3/16 cases no acute episode of encephalopathic decompensation had been noted prior to the development of the movement disorder. Baseline BMFDRS motor score ranged from 58.5–114, median 105, with a GMFCS-equivalence level of V for 10/16 cases, GMFCS IV for 4/15 and GMFCS II for 1/15 cases, demonstrating the severity of movement disorder in these CAYP.

Surgery was well tolerated in all cases, with no metabolic complications encountered in the peri-operative period for any CAYP.

### Imaging findings

**Fig. 1:**
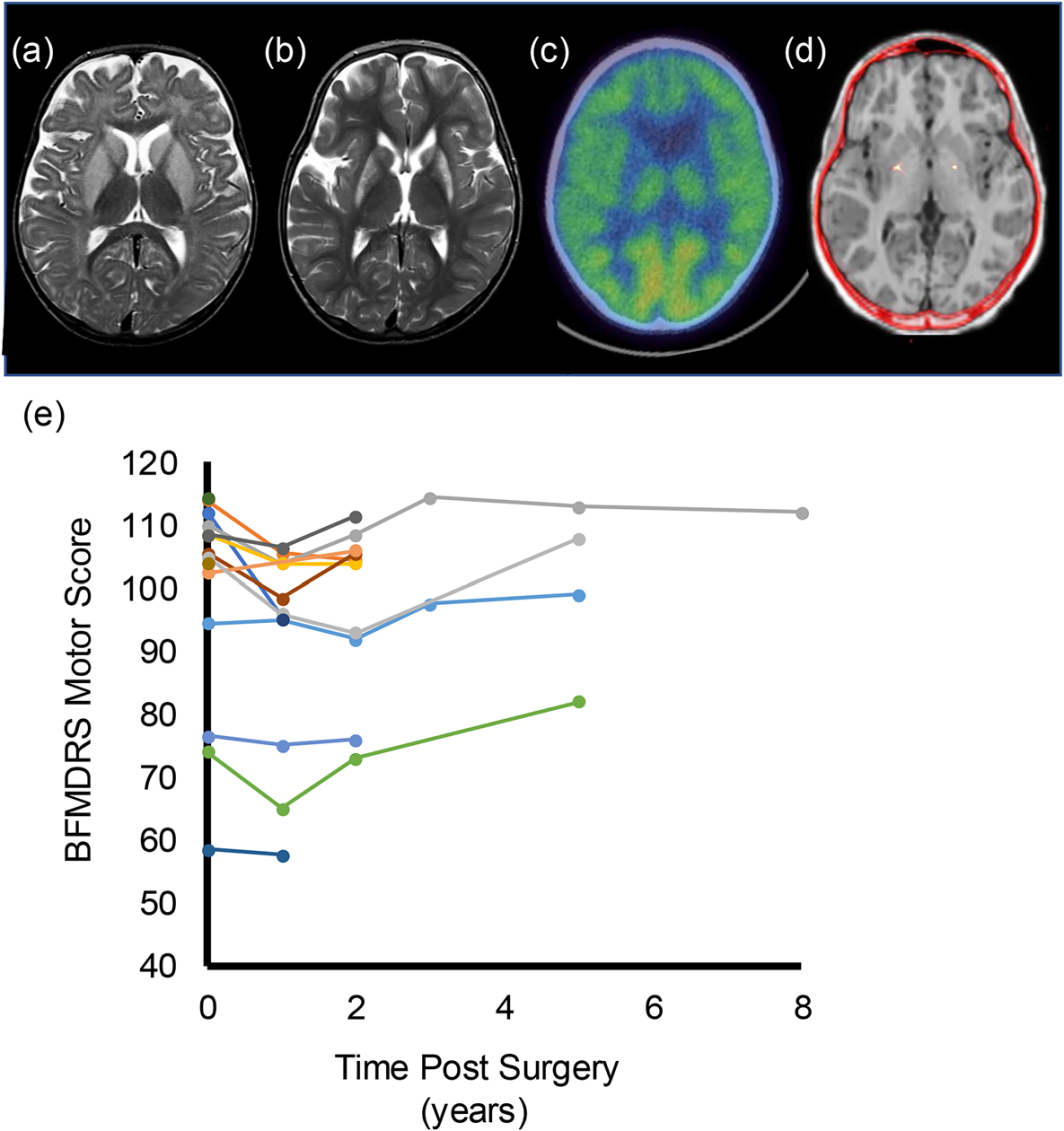
**a**–**d** Imaging from a single patient (Case 15). **a** Initial MRI imaging at the time of decompensation at 7 months of age, with bilateral basal ganglia swelling. **b** MRI imaging prior to DBS surgery at 3 years showing loss of volume and gliosis in the previously swollen parenchyma. **c** FDG-PET prior to surgery demonstrating severely reduced uptake in the pallido-putaminal complex. **d** Co-registration of post-operative CT imaging to pre-operative MRI demonstrating DBS electrode position. **e** Changes in Burke–Fahn–Marsden Dystonia Rating Scale Motor Score (BFMDRS) (*Y*-axis) for each individual CAYP compared to time following surgery (*x*-axis)

**Fig. 2:**
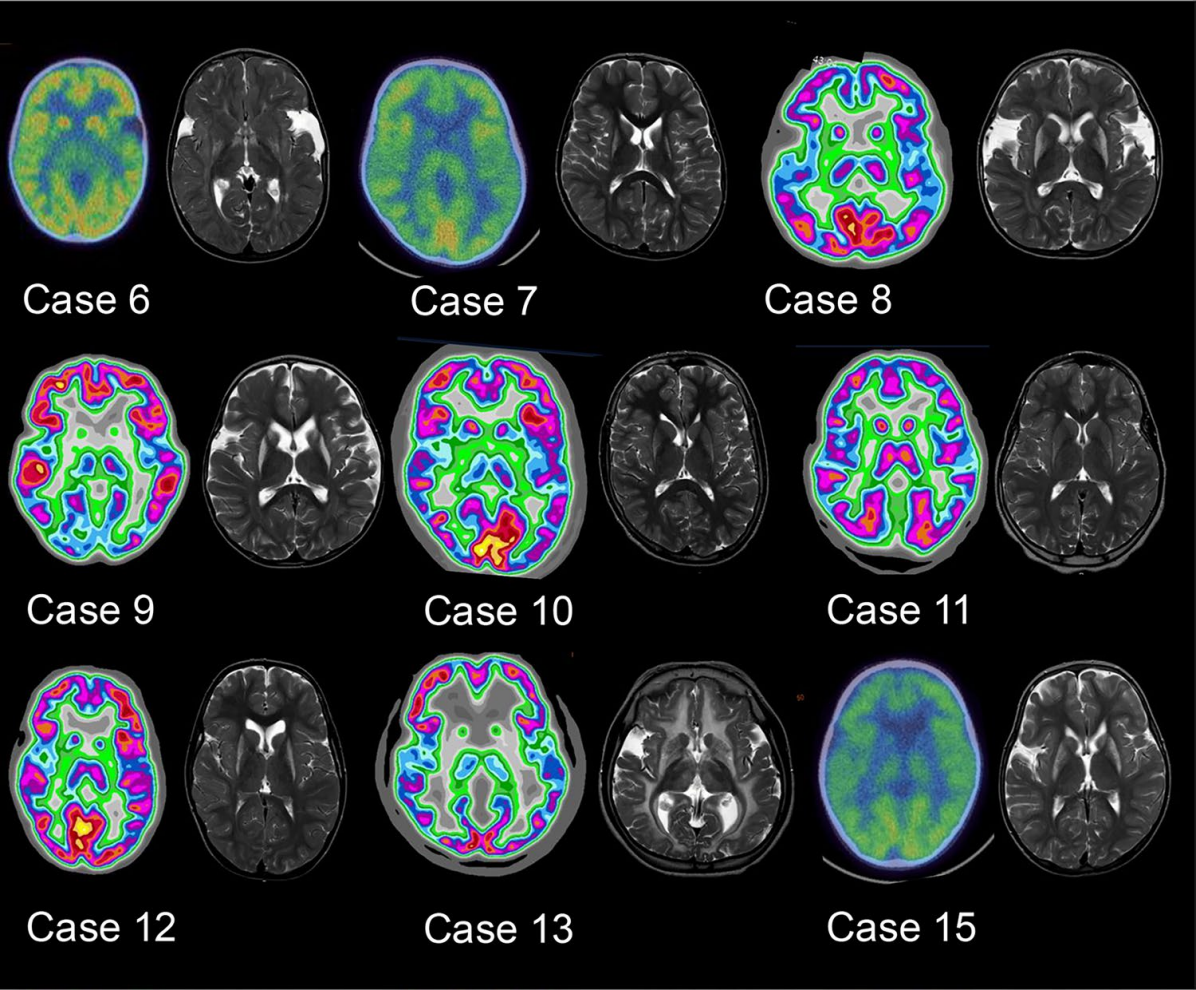
Comparison of FDG-PET and T2-weighted MRI images for Cases 6–13 and Case 14 (FDG-PET images could not be retrieved for Cases 1–5, though reports were available, and were not obtained in Cases 14 and 16). A summary of abnormalities identified for each case is provided in Table 2

Structural MRI imaging was available for review in all CAYP operated at the ELCH, with FDG-PET available in 14/15 CAYP (Table 2). In all cases abnormalities of the deep grey nuclei were bilateral in nature (i.e. unilateral structural changes were not observed). Structural abnormalities and reduced or absent FDG PET imaging glucose uptake in the putamen was seen in all 14 CAYP with FDG PET. Structural abnormalities were also identified in the pallidum (14/15 CAYP), the caudate (11/15 CAYP), the thalamus (4/15 CAYP), and in the white matter in 12/15 CAYP. Abnormal FDG uptake in the caudate was seen in 4/14 CAYP (all of whom also demonstrated structural MRI lesions within the caudate), and abnormalities of FDG uptake in the thalamus was seen in 2 CAYP (1 of whom did not demonstrate structural changes in either thalamus). Examples of progression in MRI findings and FDG-PET images for a single case are illustrated by Fig. 1a. A comparison of FDG-PET and MRI findings for Cases 6–15 is shown in Fig. 2.

**Table 2:** Summary of imaging and neurophysiological findings

Case number	FDG-PET			MRI imaging					Neurophysiology	
	Putamen	Caudate	Thalamus	Putamen	Pallidum	Caudate	Thalamus	White matter	CMCT	SEP
1	Absent	Absent	Reduced	Abnormal	Abnormal	Abnormal	Abnormal	Abnormal	Normal	Not performed
2	Reduced	Normal	Normal	Abnormal	Abnormal	Normal	Abnormal	Abnormal	Normal	Not performed
3	Reduced	Normal	Normal	Abnormal	Abnormal	Abnormal	Normal	Abnormal	Normal	Not performed
4	Reduced	Normal	Normal	Abnormal	Normal	Normal	Normal	Normal	Normal	Not performed
5	Reduced	Normal	Normal	Abnormal	Abnormal	Abnormal	Normal	Abnormal	Normal	Normal
6	Reduced	Normal	Reduced	Abnormal	Abnormal	Normal	Normal	Abnormal	Normal	Normal
7	Reduced	Reduced	Normal	Abnormal	Abnormal	Abnormal	Normal	Abnormal	Normal	Normal
8	Reduced	Normal	Normal	Abnormal	Abnormal	Abnormal	Normal	Abnormal	Abnormal	Normal
9	Reduced	Reduced	Normal	Abnormal	Abnormal	Abnormal	Normal	Abnormal	Normal	Normal
10	Reduced	Normal	Normal	Abnormal	Abnormal	Abnormal	Normal	Normal	Normal	Abnormal
11	Reduced	Normal	Normal	Abnormal	Abnormal	Normal	Normal	Abnormal	Normal	Normal
12	Reduced	Normal	Normal	Abnormal	Abnormal	Abnormal	Normal	Abnormal	Normal	Normal
13	Absent	Normal	Normal	Abnormal	Abnormal	Abnormal	Abnormal	Abnormal	Normal	Normal
14	Not performed	Not Performed	Not Performed	Abnormal	Abnormal	Abnormal	Abnormal	Abnormal	Normal	Normal
15	Absent	Reduced	Normal	Abnormal	Abnormal	Abnormal	Normal	Normal	Abnormal	Normal

*CMCT* central motor conduction time, *FDG-PET* flurodeoxyglucose positron emission tomography, *MRI* magnetic resonance imaging, *SEP* somatosensory evoked potential

### Neurophysiological findings

Despite white matter MRI changes in all ELCH cases, CMCT measurements were only abnormal in 2/15 cases (Table 2). Notably, CMCT measurements were normal in 11/12 CAYP with white matter changes and abnormal CMCT measurements were obtained in 1 CAYP without evidence of white matter changes on MR imaging. Abnormal SSEPs where found in only 1/11 CAYP (who exhibited no abnormalities of the thalami on FDG-PET or MRI). SSEP measurements were normal in the 4 CAYP with abnormalities of the thalami on FDG-PET and/or MRI. Neurophysiological measurements were not available for Case 16.

### Outcomes following surgery

No outcome data were available for one child who moved away from the UK before 1 year post-operatively or for one child who experienced infection of the implant at 9 months post-surgery, resulting in complete removal of the implanted system. For the remaining 13 CAYP from ELCH, outcome data available ranged from 1 to 5 years post-surgery. Four CAYP had transitioned to adult services since DBS surgery. Follow-up data were available for two of these cases (Case 4 with 9 years of follow-up data at SRUH before death at the age of 30 years, and Case 5 with 3 years of follow-up data at King’s College Hospital NHS Foundation Trust, London). Death occurred prior to transition in 2 CAYP at 1.5- and 5.5-years following surgery, respectively (in neither case related to DBS). The remaining 8 CAYP continue under active follow-up at the ELCH.

BFMDRS and COPM scores were available in 12 and 11 CAYP, respectively and are shown in Table 1 and Fig. 1. BFMDRS motor score 1-year post-surgery ranged from 57.5–108.5 (median 97.25) and at last follow-up ranged from 57.5–112 (median 104). There was no statistically significant change compared to baseline at either time point, *P* > 0.05. In contrast, COPM scores demonstrated a clinically significant improvement in 7/11 CAYP at 1 year, and a clinically significant improvement in 8/11 CAYP at last follow-up. Standardised outcome measures were not available for Case 16 but whilst limited improvement in oromandibular dystonia was observed, a greater than 50% reduction in truncal and limb dystonia was estimated from clinical assessments, with significant improvement in mobility. Prior to surgery Case 16 had required multiple hospital admissions for dystonic crises, but no admissions have been required in the 9 years since DBS insertion.

A total of six complications requiring re-operation occurred in 4 CAYP during management at the ELCH (outlined in Table 1). Following transition to adult services, Case 4 required surgery to remove an infected Activa RC implant 10 years following the original surgery. Case 5 required a routine battery change 1 year following transition, complicated by infection needing repositioning of the implant after one month, and then repositioning of a “flipped” battery 1 year later. Notably, Case 16 is now 9 years following surgery in adulthood, with no revision surgeries required to date.

### Relationship between neuroimaging findings and outcome

No clear relationship between neuroimaging findings and outcome was identified. The single case with thalamic abnormalities soley on FDG-PET was amongst the 3/11 CAYP who did not show a significant improvement in COPM scores. All three of these CAYP demonstrated structural changes in all basal ganglia structures and white matter, with one child also demonstrating structural changes in the thalami.

## Discussion

This report presents multi-modal clinical, imaging and neurophysiological data from a cohort of 16 individuals with GA1, together with clinical outcomes from pallidal DBS delivered to manage their refractory dystonia. To our knowledge this is the largest GA-1 functional neurosurgery case-series of its kind.

Our key findings are (i) despite small and statistically non-significant changes in BMFDRS motor score, significant functional improvement, as measured by the COPM, was observed in 8/11 (> 70%) CAYP for whom this measure was collected, and (ii) abnormalities of basal ganglia, thalami and white matter on structural MRI or FDG-PET imaging did not preclude improvement in COPM score.

Prior to this report, outcomes following DBS had been reported for only 9 CAYP with GA1, including 3 from our centre [12]. Consistent with previous findings, only small changes in BMFDRS score were seen in our expanded cohort, with changes compared to baseline at the group level not reaching statistical significance. The BFMDRS was originally developed and validated in adults with idiopathic or genetic forms of isolated dystonia [6]. Significant limitations have been identified in the application of the BFMDRS to CAYP with acquired forms of dystonia [38], and we have previously demonstrated that CAYP may experience functional benefits following DBS surgery which are not captured by changes in BFMDRS score [18]. This is consistent with the observation of improvement in COPM in 8/11 CAYP despite minimal BFDMRS change in our cohort. Furthermore, Case 16 has experienced a sustained period free from dystonic crises since surgery. We have previously reported the application of the COPM to demonstrate improvements in individualised functional goal areas for a cohort of 30 CAYP with dystonia undergoing DBS [17]. The COPM is an evidence-based tool designed to capture self-perception of performance in everyday living over time. The reproducibility and validity of COPM has been demonstrated in a large cohort of CAYP (median age 3.7 years) [45].

Table 3 provides a summary of DBS outcomes in the 6 previously reported cases not included in our current case series. Of note, a substantial reduction in both Barry-Albright Dystonia Scale (BADS) [2] and BFMDRS score (~ 50%) was reported for one child receiving bilateral Globus Pallidus Interna DBS in combination with stimulation of the pedunculopontine nucleus [33], with similar reduction in one further child receiving bilateral pallidal stimulation [7]. Limited data are available to support the use of other neurosurgical interventions in the management of dystonia in CAYP with GA1 [42]. Intrathecal Baclofen was reported to be of benefit in a 15-year old girl with GA1, with a reduction in BADS score from 12 to 9. Her movement disorder was described as fixed and mobile dystonia and spastic quadriplegic cerebral palsy without parkinsonism [14]. Positive outcomes with the use of intraventricular baclofen have also been described in 2 patients with GA1 (aged 10 and 23 years old, with reduction in BADS score from 30.7 to 5.0 and from 29.7 to 24.3, respectively) [16], both of whom were described as exhibiting generalised dystonia. Finally, outcomes following bilateral pallidotomy have been reported for 3 cases [13, 22, 41], with reductions in BFMDRS score from 113 to 99 reported in a 12-year old [13], and from 115 to 98 in a 6-year old [22]. Qualitative improvement in dystonia was also reported for a severely affected 18-month old [41]. More formal standardised functional or non-impairment based scores have not been reported for previous cases out with our current series, consistent with our previous finding that outcome measures of interventions in childhood dystonia do not typically focus on the priorities of CAYP or their families [30].

**Table 3:** Previous reports of outcomes following Deep Brain Stimulation in patients with Glutaric Aciduria

Authors	Gender	Age at surgery	Pre-DBS BAD	Post-DBS BAD	Pre-DBS BFMDRS	Post-DBS BFMDRS	Other comment on outcome	Complications
Lipsman et al. 2010 [29]	Female	16	NA	NA	NA	NA	Mild improvement in motor symptoms	Nil reported
Air et al. 2011 [1]	NA	16.8	28	23	NA	BA	Functional improvement in left arm	Early device removal due to infection
Tsering et al. 2017 [43]	Male	16.6	21	21	49	49		Nil reported
Nerrant et al. 2018 [38]	Female	4	NA	NA	NA	NA		Details not provided
Maclean et al. 2024 [32]	Male	8	28	17	92.5	45	PPN and GPi Implantation	Nil reported
Chacón et al. 2024 [7]	Male	10	NA	NA	46	22	Upper limb choreodystonic movements improved more than lower limb dystonia	Overstimulation worsened left lower limb dystonia

*GPi* globus pallidus interna, *PPN* pedunculopontine nucleus, *NA* not available

We have previously reported normal CMCT in 50/62 children with dystonia, despite abnormal MRI imaging in 40/62 of that cohort [34]. That previous cohort included four of our currently presented CAYP with GA1. In an expanded cohort of 180 CAYP with dystonia, abnormalities in SSEP measurements were more commonly identified than changes in CMCT (47% versus 19%), with better outcomes seen following DBS when both measurements where normal [35]. Neurophysiological testing performed as part of routine pre-operative assessment for the 15 CAYP with GA1 in this cohort again identified abnormal values in only a handful of cases, despite MRI abnormalities, demonstrating the importance of a multi-modal assessment of motor and sensory pathway integrity.

Structural MR imaging in this cohort demonstrated injury of the putamen in all cases, with very common involvement also of the pallidum and caudate, and less frequent abnormalities of thalamus and white matter (Table 2). It should be noted that the patients in our cohort are a selected group within the GA1 population, all having severe, medically refractory dystonia. In this context, it is pertinent that analysis of a large cohort of MRI scans from 180 individuals with GA1 found putaminal changes to be the most reliable predictor of movement disorder [15]. In a large pattern-recognition approach to basal ganglia abnormalities in an international cohort of 305 MRI scans, GA1 was assigned on the basis of cluster analysis to a cluster of disorders with predominant T_2_-weighted hyperintensities in the striatum [37]. This cluster also included other metabolic disorders (e.g. proprionic acidaemia). Interestingly, pallidal abnormalities were a relatively infrequent finding in the cases grouped into this cluster [37], in contrast to the relatively high frequency with which they were seen in our cohort. Again, this could be explained in part by the selective nature of our cohort. Importantly, changes in basal ganglia and/or thalami did not preclude the potential to respond to DBS in the CAYP in our current series.

In a previous statistical analysis we have demonstrated an FDG-PET pattern of relative regional hypometabolism in the posterior putamen and pallidum as characteristic of GA1 [43]. Our qualitative analysis in the current cohort is consistent with this. As seen with structural imaging changes, hypometabolism of the basal ganglia and thalami did not preclude the possibility of a positive response to DBS.

Newborn screening for GA1 is available in most of the more economically developed countries of the world. The timing of screening varies but it is exceptionally rare for a child to have suffered striatal injury prior to screening. Screening aims to reduce the occurrence of injury from brain accumulation of glutarate and 3-hydroxyglutarate by prompt treatment of illness, prescription of l-carnitine, and a lysine-restricted, arginine-supplemented diet [5]. Despite the very clearly demonstrated benefits of this approach there remain a number of children who will develop striatal injury despite having received specialist metabolic management since newborn screening. Two biochemically distinct but clinically similar entities are recognised based on excretion of disease metabolites. Low excretors are a source of false-negatives at newborn screening but have the same risk of acute encephalopathic crisis with striatal injury [4, 20]. In addition, adherence to all aspects of this treatment strategy is challenging for families. In a review of patients screened as newborns, 32% of children did not receive treatment according to published guidelines, and overall, 30% of patients developed major motor symptoms. Despite a clear treatment effect, 7% of fully adherent patients also had serious striatal injury [4]. In certain populations where full adherence is more challenging, 90% of children experience acute encephalopathic crises despite newborn screening and implementation of full guidelines.

Striatal injury can also occur insidiously, without an apparent crisis. It has been suggested that this may be more common in children who are not fully adherent with the recommended diet [5]. It seems likely, therefore, that even with current preventative strategies the need for intervention to manage distressing dystonia will remain.

Several limitations to our present study must be acknowledged. First, this is a retrospective review, at risk of the attendant limitations of such study designs. Whilst representing a comparatively large cohort of CAYP, given the rarity of GA1 and limited access to DBS, the small number of cases precludes complex statistical analysis. SEP data were not available for all cases, nor were COPM scores. Post-operative follow-up was of limited duration, with 2/15 CAYP transitioned to adult services for whom no further data were available and 1 further CAYP having been lost to follow-up on leaving the UK. The limitations of the application of BFMDRS score in this population have been acknowledged above, further compounded by scoring performed in a clinical setting, not blinded to operative status.

## Conclusion

In this retrospective cohort, a high proportion of CAYP with GA1 undergoing pallidal DBS for refractory dystonia achieved clinically significant functional improvements as measured by the COPM, despite failure of the BFMDRS scores to demonstrate an improvement in objective dystonia measurements. This may reflect the poor sensitivity of the BFMDRS to demonstrate change in this patient group. Importantly, functional improvement was achieved in the majority of patients despite imaging evidence of damage to the basal ganglia and/or white matter. Finally, the surgical complication rate was no different from previously published data from children undergoing DBS with a broader range of aetiologies. DBS may be considered as a management option for children with GA1 who have appropriately selected goals for intervention.

## Data Availability

The authors declare that data will be made available on reasonable request.
